# Multiple functions of CXCL12 in a syngeneic model of breast cancer

**DOI:** 10.1186/1476-4598-9-250

**Published:** 2010-09-17

**Authors:** Sharon A Williams, Yuka Harata-Lee, Iain Comerford, Robin L Anderson, Mark J Smyth, Shaun R McColl

**Affiliations:** 1 School of Molecular and Biomedical Science, University of Adelaide, Adelaide, South Australia, 5005 Australia; 2 Cancer Cell Biology Program, Trescowthick Laboratories, Peter MacCallum Cancer Centre, East Melbourne, Victoria 3002, Australia; 3 Cancer Immunology Program, Trescowthick Laboratories, Peter MacCallum Cancer Centre, East Melbourne, Victoria 3002, Australia

## Abstract

**Background:**

A growing body of work implicates chemokines, in particular CXCL12 and its receptors, in the progression and site-specific metastasis of various cancers, including breast cancer. Various agents have been used to block the CXCL12-CXCR4 interaction as a means of inhibiting cancer metastasis. However, as a potent chemotactic factor for leukocytes, CXCL12 also has the potential to enhance anti-cancer immunity. To further elucidate its role in breast cancer progression, CXCL12 and its antagonist CXCL12_(P2G) _were overexpressed in the syngeneic 4T1.2 mouse model of breast carcinoma.

**Results:**

While expression of CXCL12_(P2G) _significantly inhibited metastasis, expression of wild-type CXCL12 potently inhibited both metastasis and primary tumor growth. The effects of wild-type CXCL12 were attributed to an immune response characterized by the induction of CD8^+ ^T cell activity, enhanced cell-mediated cytotoxicity, increased numbers of CD11c^+ ^cells in the tumor-draining lymph nodes and reduced accumulation of myeloid-derived suppressor cells in the spleen.

**Conclusions:**

This study highlights the need to consider carefully therapeutic strategies that block CXCL12 signaling. Therapies that boost CXCL12 levels at the primary tumor site may prove more effective in the treatment of metastatic breast cancer.

## Background

CXCL12 is a highly pleiotropic chemokine, influencing a variety of biological processes through interaction with its receptors CXCR4 and CXCR7. It is particularly prominent in the context of immune responses, acting as a potent chemotactic factor for mature T cells and monocytes [1], mature dendritic cells (DC) [2], natural killer (NK) cells and NKT cells [3].

In a landmark study, CXCR4 was shown to be expressed by various human breast cancer cells, and metastasis of these cells in severe combined immunodeficient (SCID) mice could be inhibited with neutralizing CXCR4 antibodies [4]. Subsequent studies have demonstrated CXCR4 expression in a variety of cancer cells and tumors (reviewed in [5]). CXCL12 and its receptors are not only involved in processes specific to metastasis; many of their functions promote primary tumor growth. CXCL12 can promote breast tumor cell survival and proliferation, at least in immune deficient mice [6]. Further, it can induce production of matrix metalloproteinases [7], and promote invasion of breast cancer cells *in vitro *[8]. CXCL12 is also an angiogenic chemokine and CXCL12 secretion by fibroblasts co-implanted into nude mice with breast carcinoma cells promotes vascularization of the developing tumors [6].

A number of different agents have been used to block the CXCL12-CXCR4 interaction as a means of inhibiting metastasis. These include neutralizing anti-CXCR4 antibodies [4], pharmacologic inhibitors such as AMD3100 [9] and T140 [10], and small interfering RNAs directed towards CXCR4 [8,9]. In this work, we sought to determine whether a modified form of CXCL12, CXCL12_(P2G)_, which acts as an antagonist of CXCR4 [11], is able to block metastasis in the 4T1.2 orthotopic mouse model of breast cancer. Concomitantly, we examined the effects of exogenous wild-type CXCL12 expression on metastasis. We found that CXCL12_(P2G) _was able to inhibit both spontaneous and experimental metastasis, without affecting primary tumor growth. In contrast, wild-type CXCL12 blocked both metastasis and primary tumor growth in a manner that was dependent on the induction of an anti-tumor immune response.

## Results

### Generation and *in vitro *characterization of CXCL12 construct-expressing 4T1.2 mammary carcinoma cell lines

**Figure 1:**
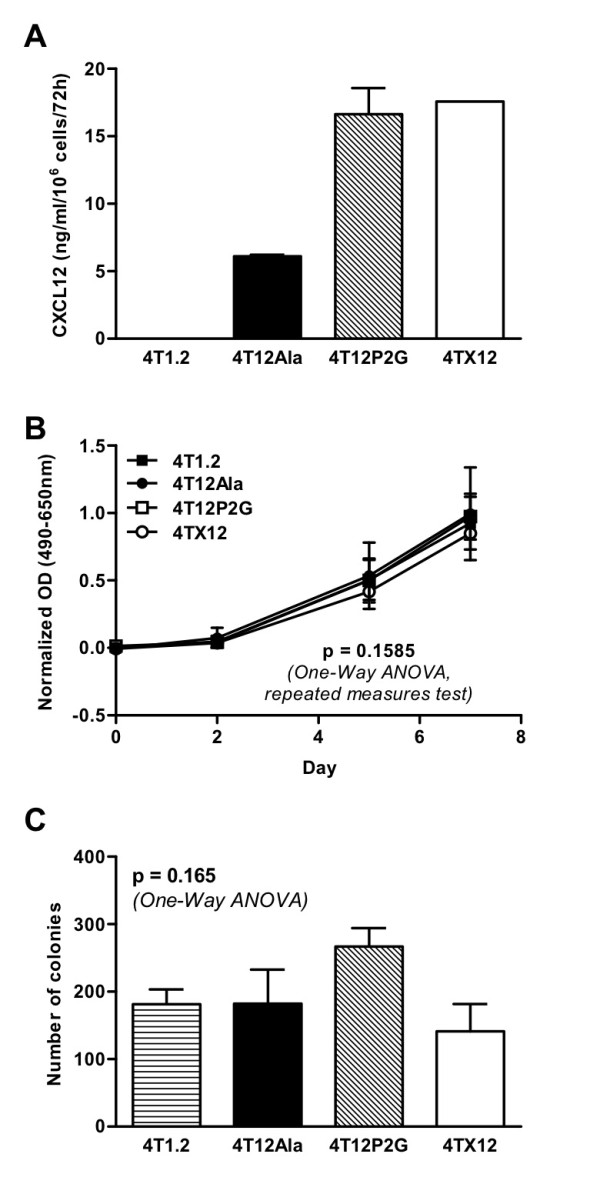
***In vitro *characterization of transfected 4T1.2 cells**. A) Expression of CXCL12 constructs by transfected cell lines as determined by CXCL12 ELISA. Note that detection of 4T12Ala protein is likely to be hampered because of the disrupted tertiary structure of the chemokine. Similar levels of expression of all three constructs were detected by PCR (Additional File 1). B) Growth of transfected 4T1.2 cell lines compared to wild-type 4T1.2 cells in medium supplemented with 1% FCS, as determined by XTT proliferation assay. Data points represent mean ± SEM of determinations from 2 independent experiments. C) Tumorigenicity of transfected 4T1.2 cells grown in semi-solid agar compared to wild-type 4T1.2 cells. Bars represent mean ± SEM of determinations from 3 experiments.

To determine if antagonizing CXCR4 by means of the CXCR4 antagonist CXCL12_(P2G) _would impact on growth and metastasis of 4T1.2 tumors, 4T1.2 cells were transfected with a DNA construct encoding CXCL12_(P2G)_. In addition, 4T1.2 cells were transfected with a construct encoding wild-type CXCL12, to determine the effect of wild-type CXCL12 on tumor growth and metastasis. As a control, a DNA construct encoding a biologically inactive form of CXCL12, CXCL12_(Ala)_, in which the four cysteine residues were mutated to alanine residues, was transfected into 4T1.2 cells. The cell lines generated following transfection of the wild-type CXCL12, CXCL12_(P2G) _and CXCL12_(Ala) _constructs were labeled 4TX12, 4T12P2G and 4T12Ala, respectively. Expression of the CXCL12 constructs was confirmed by RT-PCR (Additional File 1) and ELISA (Fig. 1A). Neither CXCL12_(P2G) _nor CXCL12 expression significantly affected the growth of 4T1.2 cells *in vitro *(Fig. 1B, C and Additional File 2).

### The effects of CXCL12_(P2G) _and CXCL12 expression on tumor growth and metastasis

**Figure 2:**
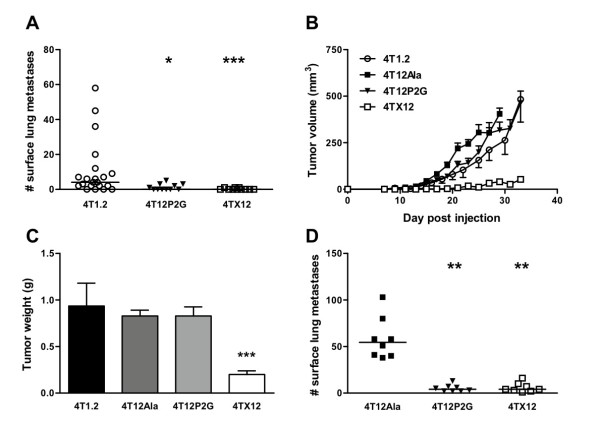
**The effect of CXCL12 and CXCL12_(P2G) _expression on growth and metastasis of 4T1.2 tumors**. A) Effects of CXCL12_(P2G) _and CXCL12 expression on the formation of spontaneous lung metastases in 4T1.2 tumor-bearing mice. Balb/c mice were injected with 4T1.2, 4T12P2G or 4TX12 cells i.m.f.p.. On day 21, lungs were removed and surface lung metastases were counted under a dissecting microscope. Bars represent the median. *, P < 0.05; ***, P < 0.001 (Kruskal-Wallis), *n *= 11-21 from two independent experiments. B) BALB/c mice were injected with 4T1.2, 4T12Ala, 4T12P2G or 4TX12 cells i.m.f.p. and tumor growth was monitored using digital calipers. C) At the end of the experiment, mice were killed and their tumors resected and weighed ***, P < 0.0001 (ANOVA), *n *= 12. Data points and bars represent mean ± SEM. D) Effects of CXCL12_(P2G) _and CXCL12 expression in 4T1.2 cells on the formation of experimental lung metastases. BALB/c mice were injected intravenously with 7.5 × 10^5 ^cells in PBS. After approximately 2 weeks, lungs were harvested and surface metastatic nodules were enumerated under a dissecting microscope. Data points represent the number of surface metastatic nodules of individual mice. Bars represent the median. **, P < 0.01 (Kruskal-Wallis).

**Figure 3:**
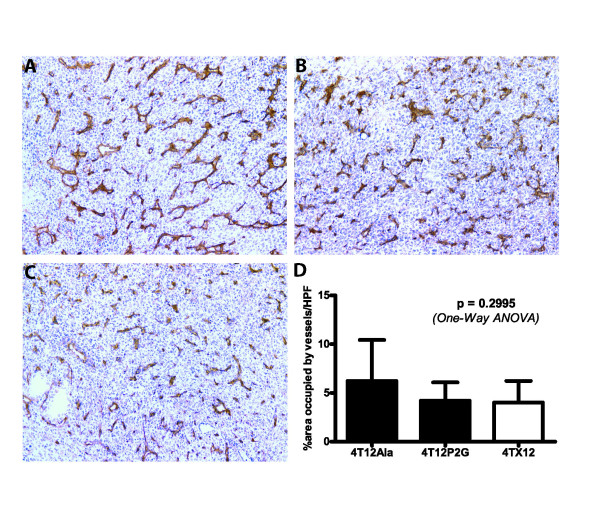
**The effect of CXCL12_(P2G) _and CXCL12 expression on tumor angiogenesis**. Representative micrographs of blood vessels in A) a 4T12Ala tumor, B) a 4T12P2G tumor and C) a 4TX12 tumor. Magnification: 100x. D) Quantitation of the area occupied by blood vessels. The percentage area occupied by blood vessels per high power field was determined using ImageJ software (http://rsbweb.nih.gov/ij/). Bars represent the mean ± SD, *n *= 5-12 individual mice.

In our initial experiments, we found that CXCL12_(P2G) _significantly inhibited spontaneous metastasis of 4T1.2 tumors to the lungs of tumor-bearing mice (Fig. 2A), without affecting primary tumor growth (Figs. 2B, C). Migration of CXCL12_(P2G)_-expressing 4T12P2G cells to the lungs was also inhibited when the tumor cells were introduced directly into the circulation (Fig. 2D), suggesting that CXCL12_(P2G) _interferes with steps of metastasis that occur after metastatic cells have escaped from the primary tumor. Wild-type CXCL12 similarly inhibited both spontaneous and experimental metastasis, but in contrast to CXCL12_(P2G)_, CXCL12 concomitantly inhibited primary tumor growth (Fig. 2). The inhibitory effects of CXCL12 on primary tumor growth were confirmed in the EMT6.5 syngeneic mouse model of breast cancer (Additional File 3). Neither CXCL12_(P2G) _nor CXCL12 expression affected tumor angiogenesis (Fig. 3).

### CXCL12 expression promotes an anti-tumor immune response

The ability of wild-type CXCL12 to inhibit both primary tumor growth and metastasis makes it a more attractive candidate than the CXCR4 antagonist for incorporation into therapeutic strategies for breast cancer. Thus, we chose to investigate further this form of the chemokine to determine how it might exert its effects. Since CXCL12 expression did not affect the growth of tumor cells *in vitro*, it seemed likely that inhibition of tumor growth could be due to the effects of CXCL12 on the host rather than on the tumor cells directly. Given that CXCL12 induces responses in a variety of leukocytes, we sought to determine whether CXCL12 was affecting the anti-tumor immune response.

**Figure 4:**
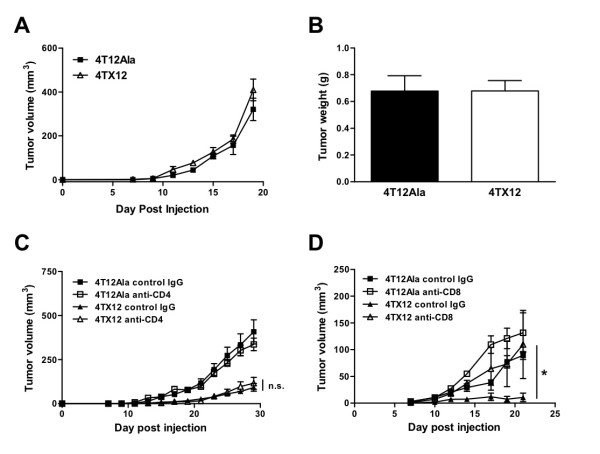
**Growth of 4TX12 tumors in immunodeficient and cell subset-depleted mice**. A-B) SCID mice were injected i.m.f.p. with 1 × 10^5 ^cells. Tumor volume (mean ± SEM; A) and final tumor weight (mean ± SEM; B) were measured (*n *= 5). C-D) BALB/c mice were injected i.m.f.p. with 1 × 10^5 ^cells and treated with anti-CD4, anti-CD8, control IgG or PBS as described in Materials and Methods. Data points represent mean tumor volume ± SEM in mice depleted of (C) CD4^+ ^cells, *n *= 8, and (D) CD8^+ ^cells, *n *= 6; *, *p *< 0.05; ns, not significant compared to 4TX12 tumors in mice treated with control IgG (t test).

**Figure 5:**
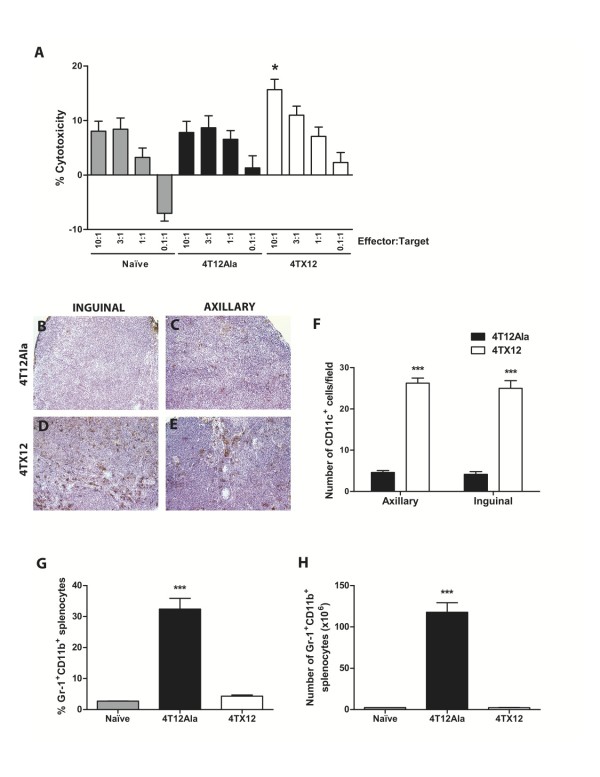
**The effect of CXCL12 on cell-mediated cytotoxicity and the accumulation of dendritic cells and MDSC**. A) Cell-mediated cytotoxicity against 4T1.2 tumors. BALB/c mice were injected i.m.f.p. with 1 × 10^5 ^cells. After approximately 5 weeks, effector cells were prepared for use in a cytotoxicity assay as described in Materials and Methods. *, P < 0.05, significantly different from naïve effectors and effectors from 4T12Ala tumor-bearing mice (Kruskal-Wallis). B-F) CD11c^+ ^cell infiltration of tumor-draining lymph nodes. Cryostat sections from inguinal (B, D) and axillary (C, E) draining lymph nodes 6 days post 4T12Ala (B, C) or 4TX12 (D, E) cell injection were stained with anti-CD11c antibody. Magnification: 100×. F) CD11c^+ ^cells were enumerated in ten randomly selected high power fields per lymph node. Bars represent the mean number (± SEM) of CD11c^+ ^cells per field. *n *= 3 mice (i.e. 6 lymph nodes) per group from two independent experiments. ***, p < 0.0001 (t test). G-H) Accumulation of MDSC in spleens of tumor-bearing mice. BALB/c mice were injected i.m.f.p. with 1 × 10^5 ^cells. Spleens were resected 28 days later and the percentage (G) and the absolute numbers (H) of Gr1^+^CD11b^+ ^splenocytes in tumor-bearing and control (naïve) mice was determined by flow cytometry. Bars represent the mean ± SEM of duplicate determinations from 5 mice. ***, P < 0.0001 (t test).

A variety of gene-targeted and cell subset-depleted mice were used to characterize the immune response to 4T1.2 tumors. In SCID mice, the growth inhibition of CXCL12-expressing 4TX12 tumors was completely lost (Fig. 4A, B). Subsequent cell depletion experiments in immune competent mice revealed that CD8^+^, but not CD4^+ ^cells were required for the anti-tumor effect (Fig. 4C, D), suggesting that cytotoxic T cells play an important role in CXCL12-mediated tumor inhibition. Accordingly, the *in vitro *cytotoxic response of effector cells derived from 4TX12 tumor-bearing mice was enhanced compared to that of control tumor-bearing mice (Fig. 5A). Moreover, the anti-tumor effect of CXCL12 was reduced in pfp^-/- ^mice, which lack the cytotoxic mediator perforin, and TRAIL^-/- ^mice (Table 1). Since IFN-γ production is another major function of effector CD8^+ ^T cells [12], we also assessed the growth of 4TX12 tumors in IFN-γ^-/- ^mice. As shown in Table 1, IFN-γ is important for the inhibition of 4T1.2 tumor growth mediated by CXCL12.

**Table 1:** CXCL12-mediated inhibition of tumor growth in different strains of mice

Strain	Experiment	Day terminated	Mean tumor weight (g) ± SEM (*n*)		*p *value (t test)	4TX12 tumor weight as% of control
					
			4T12Ala	4TX12		
Wild type	1	33	0.83 ± 0.06 (*12*)	0.20 ± 0.04 (*12*)	***, <0.0001	24.2
	2	27	0.90 ± 0.13 (*5*)	0.21 ± 0.07 (*6*)	***, 0.0008	22.9
SCID	1	19	0.56 ± 0.11 (*5*)	0.60 ± 0.02 (*5*)	ns, 0.7426	106.6
	2	23	0.79 ± 0.08 (*5*)	0.76 ± 0.06 (*5*)	ns, 0.7236	95.4
pfp^-/-^	1	28	0.79 ± 0.10 (*6*)	0.49 ± 0.05 (*6*)	*, 0.0232	61.4
TRAIL^-/-^	1	28	0.78 ± 0.14 (*7*)	0.44 ± 0.03 (*7*)	*, 0.0369	57.1
IFN-γ^-/-^	1	28	1.21 ± 0.17 (*7*)	0.64 ± 0.18 (*7*)	*, 0.0403	52.9

*ns*, not significant.

Examination of the secondary lymphoid organs of tumor-bearing mice revealed some significant differences. The tumor-draining lymph nodes (DLN) from 4TX12 tumor-bearing mice harbored a greater number of CD11c^+ ^DC than those from controls (Fig. 5B-F), in agreement with a previous study in colon carcinoma [13]. In the spleen, we found that myeloid-derived suppressor cells (MDSC), a population of Gr1^+^CD11b^+ ^immature myeloid progenitors that have profound immunosuppressive effects in malignant disease [14], were significantly reduced in 4TX12 tumor-bearing mice compared to control tumor-bearing mice (Fig. 5G-H). Together these data indicate that the pro-immunogenic effects of CXCL12 are two-fold: first it promotes the accumulation of cells that activate the immune response, and second, it limits the generation of cells with immunosuppressive effects.

## Discussion

In this study we have shown that a novel antagonist of CXCR4, a mutant form of the natural ligand, CXCL12, is capable of blocking the spontaneous metastasis of breast cancer cells from the primary tumor in an orthotopic mouse model of breast cancer. Furthermore, we have demonstrated that blockade of CXCR4 at metastatic steps that follow entrance of malignant cells into the circulation is sufficient to reduce metastasis, although we cannot rule out a role for CXCR4 in escape from the primary tumor. Several studies have reported the inhibition of metastasis of human MDA-MB-231 breast cancer cells in SCID mice using anti-CXCR4 antibodies [4,15], siRNA [8,16] or peptide antagonists of CXCR4 [10,15]. Our data extend these findings by demonstrating that blockade of CXCR4 inhibits both spontaneous and experimental metastasis in a more biologically relevant orthotopic model of breast cancer. Our results are in agreement with those obtained in a similar study by Smith et al [9] in which siRNA knockdown of CXCR4 inhibited both spontaneous and experimental metastasis. However, in that study, CXCR4 knockdown was also found to inhibit primary tumor growth. We found that blocking CXCR4 by expressing CXCL12_(P2G) _did not alter primary tumor growth, demonstrating the metastasis-specific effect of this antagonist.

In contrast to CXCL12_(P2G)_, wild-type CXCL12 inhibited both metastasis and primary tumor growth. It has been demonstrated previously that that co-implantation of tumor cells with fibroblasts expressing CXCL12 enhances tumor growth [6]. However, that study was performed in T cell-deficient nude mice and thus our findings in an immunocompetent mouse model are not contradictory. Moreover, although Orimo et al found that the enhanced tumor growth was in part dependent on increased angiogenesis [6], we found that neither expression of CXCL12 nor CXCL12_(P2G) _altered tumor angiogenesis in our model. Similarly, Smith et al did not see inhibition of angiogenesis when they knocked down CXCR4 expression in 4T1 cells [9]. It has been shown that 4T1 cells produce endogenous vascular endothelial growth factor (VEGF) [17] but not CXCL12 (Fig. 1A and Additional File 1). Thus it is not surprising that CXCL12 and CXCL12_(P2G) _expression does not affect angiogenesis in this model since the tumor cells are capable of producing VEGF independently of CXCL12. The anti-tumor effects of CXCL12 and anti-metastatic effects of CXCL12_(P2G) _are therefore unlikely to be a result of reduced angiogenesis.

Further analysis of the anti-tumor effect of CXCL12 revealed that it was dependent on the presence of functional CD8^+ ^T cells, in agreement with a previous study that demonstrated the importance of CD8^+ ^T cells in CXCL12-mediated growth inhibition of melanoma and lung carcinoma [13]. However, our findings contrast with those in a model of leukemia, in which CD4^+^, but not CD8^+^, T cells were required [18] and the findings in a fibrosarcoma model, where both T cell subsets were necessary [19]. Taken together, these data suggest that CXCL12 is able to induce different anti-tumor responses depending on the tumor type.

In line with the requirement for CD8^+ ^T cells, the *in vitro *cytotoxic response of effector cells derived from 4TX12 tumor-bearing mice was enhanced compared to that of control tumor-bearing mice. In support of this finding, the anti-tumor effect of CXCL12 was reduced in pfp^-/- ^mice and in TRAIL^-/- ^mice, suggesting that the CD8^+ ^T cells exert their effects via the death receptor pathway as well as the granule exocytosis pathway. IFN-γ was also found to be important for CXCL12-mediated inhibition of 4T1.2 tumor growth which indicates that CD8^+ ^T cells may exert not only direct cytotoxic function against tumor cells, but also indirect effects via IFN-γ-mediated activation of other effector cells.

Despite the requirement for functional CD8^+ ^T cells for the tumor inhibitory effect of CXCL12, increased infiltration of CXCL12-expressing tumors by T cells was not observed (data not shown). However, the number of dendritic cells in tumor-DLN was increased, in agreement with a previous study in colon carcinoma [13]. Based on the observations discussed above, it seems likely that CXCL12, while not increasing T cell infiltration of the tumor mass, acts to enhance T cell activation by increasing the proportion of DC in the tumor-DLN. Responsiveness to CXCL12 increases during the maturation process of DC [2], and CXCR4 directly promotes survival of DC, particularly those of a mature phenotype [20]. The ability of CXCL12 to enhance the survival of mature DC may be critical to the generation of sufficient mature DC for priming of anti-tumor T cells and may represent a key aspect of the anti-tumor response. Although previous work by Zou et al [21] has shown that CXCL12 expression by ovarian tumors promotes the accumulation of immunosuppressive plasmacytoid DCs, we did not observe this in our model. As discussed above, CXCL12 can induce different responses depending on the tumor type. In addition, that study was performed in human tissues expressing endogenous CXCL12, in contrast to our study in which CXCL12 was overexpressed. The levels of CXCL12 at the tumor site may determine the type of immune response generated, with lower levels promoting the accumulation of immunosuppressive cells while higher levels recruit immunoresponsive cells.

Another significant finding of this work was that CXCL12 expression prevented the accumulation of MDSC in the spleens of tumor-bearing mice. It has been shown previously that mice bearing 4T1 tumors have elevated levels of splenic MDSC, and that reduction of MDSC and resistance to metastatic disease following removal of the primary tumor is dependent on the presence of CD8^+ ^cells and IFN-γ [17]. In our model, it is possible that induction of CD8^+ ^T cell activity and IFN-γ production by tumor-derived CXCL12 drives differentiation of MDSC into mature macrophages, abrogating their immunosuppressive effects. Such a mechanism has been demonstrated in a model of lung cancer [22].

## Conclusions

Our studies demonstrate a significant anti-tumor role for the CXCL12-CXCR4 axis in breast cancer progression. Early high-level CXCL12 expression at the tumor site induces an anti-tumor response that depends on CD8^+ ^T cells. CXCL12 expression results in enhanced cell-mediated cytotoxicity towards tumor cells, increased accumulation of CD11c^+ ^cells in the TDLN and reduced accumulation of MDSC in the spleen. In addition, high levels of CXCL12 in the tumor microenvironment may disrupt the CXCR4 driven chemotactic response of tumor cells towards CXCL12 expressing tissues such as bone and lung. Although antagonism of CXCR4 is an effective means of inhibiting tumor metastasis, our results highlight the need to consider carefully the merit of using such a strategy as a therapy for breast cancer. It is noteworthy that a recent study has shown that expression of CXCL12 in breast cancer tissues significantly correlates with disease-free and overall survival [23]. Thus therapies that incorporate boosting of CXCL12 levels at the primary tumor site may be a more holistic treatment of metastatic breast cancers by enhancing the anti-tumor immune response.

## Methods

### Antibodies and reagents

The following reagents were used in this study: rat anti-CD31, biotinylated rat anti-Gr1, biotinylated hamster anti-CD11c and PE-Cy7-conjugated rat anti-CD11b (BD Biosciences, San Jose, CA); streptavidin-conjugated phycoerythrin, fluorescein isothiocyanate and horseradish peroxidase (HRP), HRP-conjugated donkey anti-rat IgG and mouse gamma globulin (Rockland, Gilbertsville, PA). Depleting anti-CD4, -CD8 and control IgG were produced from the GK1.5, 53-6.72 and MAC4 hybridomas, respectively.

### Cells

The mouse breast cancer cell lines 4T1.2 (derived from a spontaneous mammary carcinoma in a Balb/c mouse [24]) and EMT6.5 (derived from EMT6 [25]) were cultured in Alpha MEM and DMEM (Invitrogen, Carlsbad, CA), respectively. Media were supplemented with 10% fetal calf serum and 1% penicillin/gentamycin solution ("complete media").

### CXCL12 DNA constructs

cDNAs encoding wild-type mouse CXCL12, CXCL12_(P2G) _(a CXCR4 antagonist in which the second proline residue of wild-type CXCL12 is mutated to glycine) and CXCL12_(Ala) _(a putative inactive form of CXCL12 in which the mutations Cys9Ala and Cys11Ala disrupt the tertiary structure of the molecule) with 6His carboxyl tags were cloned into the pEF-IRES-puro6 vector (a gift from Dr Dan Peet, University of Adelaide).

### Transfections

The 4T1.2 cell line was transfected by electroporation. Clones of stably transfected cells (selected with puromycin) were obtained by limiting dilution. Equal numbers of cells from each of the five highest secreting clones were pooled for further experiments. Expression of the CXCL12 protein was confirmed by ELISA (Fig. 1A).

### Measurement of CXCL12 construct mRNA expression

Total cell RNA was extracted using TRIzol^® ^reagent (Invitrogen) according to the manufacturer's instructions and treated with RNase-free DNase (1 U/μl; Promega, Madison, WI). A total of 5 DNase-treated RNA was reverse transcribed using random hexamers (Promega) and the reverse-transcriptase Superscript III (200 U/μl; Invitrogen), as directed by the manufacturer. The cDNA obtained was treated with DNase-free RNase A (10 mg/ml; Sigma, St Louis, MO) for 1 hour at 37°C, and 1.25μl was used as template in subsequent PCR reactions. The sequences of the primers (synthesised at Geneworks, Adelaide, Australia) used for PCR amplification were mCXCL12 F: 5'-GGAATTCGCCACCATGGACGCCAAGGTCGTCG-3', mCXCL12 R: 5'-GACTAGTTCAGTGATGGTGATGGTGGTGCATCTTGAGCCTC-3', mGAPDH F: 5'-TCCTTGGAGGCCATGTAGGCCAT-3' and mGAPDH R: 5'-TGATGACATCAAGAAGGTGGTGAAG-3'. The reaction was carried out in a final volume of 25 μl and contained 5 μl F primer (5 μM), 5 l R primer (5 μM), 2.5 l 10 × Mg^2+^-free EXT buffer and 1.25 l MgCl_2 _(50 μM), 1 l dNTPs (10 μM), 0.25 l DyNAzyme EXT polymerase and 1.25 μl of template cDNA. The reaction was carried out on a Programmable Thermal Controller under the following conditions: 94°C for 2 minutes, then 35 cycles of 94°C for 30 seconds, 52°C for 45 seconds and 72°C for 1 minute, followed by a final extension step of 72°C for 10 minutes. Fragments were visualized by electrophoresis on a 2% agarose gel.

### Measurement of secreted CXCL12 protein

Cell culture supernatants were collected from 5 × 10^6 ^cells cultured for 3 days and protease inhibitor cocktail (Sigma) was added at a 1:200 dilution. An ELISA plate (Costar, Corning, NY) coated overnight with 2 g/ml anti-CXCL12 capture antibody (R&D Systems, Minneapolis, MN) was blocked with PBS containing 1% BSA (Sigma) and 5% sucrose before the addition of the culture supernatants. Biotinylated anti-CXCL12 antibody (R&D Systems) at 200 ng/ml followed by streptavidin-HRP (Rockland) diluted 1:20000 were used to detect bound CXCL12. Colorimetric quantitation was performed using an *o*-phenylenediamine kit (Sigma) as per the manufacturer's instructions and absorbance was measured at 490 nm.

### *In vitro *proliferation assay

Cells (2.5 × 10^3^/ml) were cultured in medium supplemented with 0.1%, 1% and 10% fetal calf serum for up to 7 days. At days 0, 2, 5 and 7, sodium 3'-[1-(phenylaminocarbonyl)-3,4-tetrazolium]-bis (4-methoxy-6-nitro) benzene sulfonic acid hydrate (XTT; Sigma) was used to determine the relative number of viable cells per well according to the manufacturer's instructions.

### Soft agar cloning

Iscove's Modified Dulbecco's Medium (IMDM; Invitrogen) was prepared containing 10% FCS, penicillin/gentamycin/Fungizone (Invitrogen) and Bacto Agar (BD Diagnostic Systems, Sparks, MD). The wells of a 6-well plate were coated with 1 ml IMDM + 0.7% Agar, which was then allowed to set. A suspension of 1 × 10^3 ^cells in 1 ml IMDM + 0.3% agar was overlaid into the wells and allowed to set at room temperature. Plates were incubated at 37°C in 5% CO_2 _for 10 days and colonies formed were counted under a dissecting microscope.

### Tumor growth

Female 6-8 week old BALB/c mice were bred and maintained at the University of Adelaide Animal Facility. SCID mice were obtained from the Animal Resources Centre (Canning Vale, WA, Australia). TRAIL^-/-^, pfp^-/- ^and IFN-γ^-/- ^mice on the BALB/c background (backcrossed at least 10 generations) were bred at the Peter MacCallum Cancer Centre [26]. Tumor cells (1 × 10^5 ^in 10 μl PBS) were injected into the fourth mammary fat pad (i.m.f.p.). Tumor dimensions were determined with digital calipers and volume calculated using the equation width^2 ^× length/2. For depletion experiments, mice also received 100 μg of depleting antibody by intraperitoneal injection the day before, on the day of receiving cells and weekly thereafter. Depletion of cell subsets was confirmed by flow cytometric analysis of splenocytes.

### Measurement of metastasis

For measurement of spontaneous metastasis, tumor cells (1 × 10^5 ^in 10 μl PBS) were injected into the fourth mammary fat pad. Approximately 4 weeks later, mice were killed and their lungs were inflated with PBS containing 85% India ink, then fixed in 4% formaldehyde. Surface lung metastases were counted under a dissecting microscope. For measurement of experimental metastasis, 7.5 × 10^5 ^cells in PBS were injected into the tail vein. After approximately 2 weeks, lungs were harvested and fixed in 4% formaldehyde. Surface lung metastases were counted under a dissecting microscope.

### Cytotoxicity assay

Mice received 1 × 10^5 ^cells by mammary fat pad injection. After 5 weeks, spleens and axillary lymph nodes from each mouse were harvested as a source of effector cells. Target 4T1.2 cells were labeled with 0.2 μM calcein AM (Invitrogen), plated in complete RPMI at 2.5 × 10^4 ^cells/well in a 96-well plate and allowed to adhere 3 hours at 37°C. Effector cells (depleted of red blood cells) were then added at the indicated concentrations. Plates were incubated at 37°C in 5% CO_2 _for four days. Non-adherent cells were removed and fluorescence of the remaining cells was measured on a Molecular Imager FX (BioRad Laboratories, Hercules, CA). Percentage cytotoxicity was calculated as 100-(target fluorescence/target only fluorescence × 100).

### Immunohistochemistry

Cryostat sections were fixed in 60% acetone/40% methanol for 10 minutes. Slides were rehydrated in phosphate buffered saline (PBS), and endogenous peroxidase activity was blocked with 0.3% hydrogen peroxide for 10 minutes. Sections were blocked with 100 μg/ml mouse gamma globulin for 30 minutes. Sections were incubated with primary antibody or isotype control for 2 hrs in a humidified chamber at room temperature, then with HRP-conjugated secondary antibody (for unconjugated primary antibody) or streptavidin-HRP (for biotinylated primary antibody) for 1 hr. Bound antibody was detected with 3,3'-diaminobenzidine (DAKO, NSW, Australia). Sections were counterstained with hematoxylin.

### Flow cytometry

Cells in PBS/1% BSA/0.04% sodium azide (PBA) were blocked with 20 μg/ml mouse gamma globulin for 20 minutes, then incubated with primary antibodies or isotype-matched controls for 30 minutes. Primary antibodies were detected with streptavidin-fluorochrome conjugate and LIVE/DEAD stain (Invitrogen) was used to distinguish non-viable cells.

### Statistical analysis

Statistical analysis was performed using GraphPad Instat version 5.02 (GraphPad Software, http://www.graphpad.com).

## Competing interests

The authors declare that they have no competing interests.

## Authors' contributions

SAW generated and carried out the experiments with the 4T1.2 cell lines, performed the CD4 and CD8 depletions, cytotoxicity assay and IHC and drafted the manuscript. YH generated and carried out the experiments with the EMT6.5 cell lines, performed the CD8 depletions and the MDSC experiments and participated in the analysis of the CD11c IHC. IC participated in MDSC experiments. RLA and MJS participated in the design of the study. SRM conceived of the study, and participated in its design and coordination and helped to draft the manuscript. All authors read and approved the final manuscript.

## Supplementary Material

Additional file 1 **CXCL12 construct expression in transfected 4T1.2 cells**. RNA from each of the cell lines derived from the pooled clones was reverse-transcribed and subjected to PCR. (A) CXCL12. *M: *DNA size markers, *lane 1: *4T1.2, *lane 2: *4T12Ala, *lane 3: *4TX12, *lane 4: *4T12P2G, *lane 5: *positive control CXCL12::pEF-IRES-puro6 plasmid, *lane 6: *negative control (no template in PCR reaction). (B) GAPDH. *M: *DNA size markers, *lane 1: *4T1.2, *lane 2: *4T12Ala, *lane 3: *4TX12, *lane 4: *4T12P2G, *lane 5: *negative control (no reverse transcriptase), *lane 6: *negative control (no template in reverse transcription reaction), *lane 7: *negative control (no template in PCR reaction). Click here for file

Additional file 2 **The effect of CXCL12 expression on *in vitro *proliferation of 4T1.2 cells in complete medium and serum-reduced medium**. Growth of transfected 4T1.2 cell lines as determined by XTT proliferation assay compared to wild-type 4T1.2 cells. (A) Cells grown in medium supplemented with 0.1% FCS. (B) Cells grown in medium supplemented with 10% FCS. Data points represent mean ± SEM of determinations from 2 independent experiments. Click here for file

Additional file 3 **The effect of CXCL12 expression on growth of EMT6.5 tumors**. BALB/c mice were injected with EMTX12 or EMT12Ala cells i.m.f.p. and tumor volume was monitored (A). At the end of the experiment, mice were killed and their tumors resected and weighed (B). **, P < 0.005 (t test), *n *= 10. Data points and bars represent mean ± SEM. Click here for file
