# Supplementary figures and images for: Multiple functions of CXCL12 in a syngeneic model of breast cancer
Source: Mol Cancer. 2010 Sep 17;9:250. doi: 10.1186/1476-4598-9-250 (PMC3098012; doi:10.1186/1476-4598-9-250)

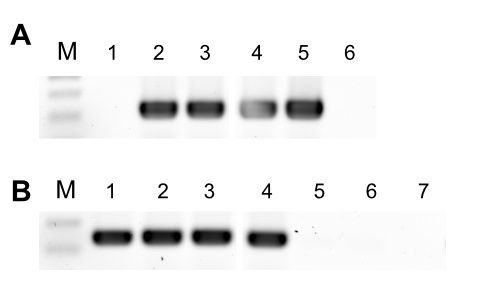

Supplement: Additional file 1 — CXCL12 construct expression in transfected 4T1.2 cells. RNA from each of the cell lines derived from the pooled clones was reverse-transcribed and subjected to PCR. (A) CXCL12. M: DNA size markers, lane 1: 4T1.2, lane 2: 4T12Ala, lane 3: 4TX12, lane 4: 4T12P2G, lane 5: positive control CXCL12::pEF-IRES-puro6 plasmid, lane 6: negative control (no template in PCR reaction). (B) GAPDH. M: DNA size markers, lane 1: 4T1.2, lane 2: 4T12Ala, lane 3: 4TX12, lane 4: 4T12P2G, lane 5: negative control (no reverse transcriptase), lane 6: negative control (no template in reverse transcription reaction), lane 7: negative control (no template in PCR reaction). [file 1476-4598-9-250-S1.JPEG]

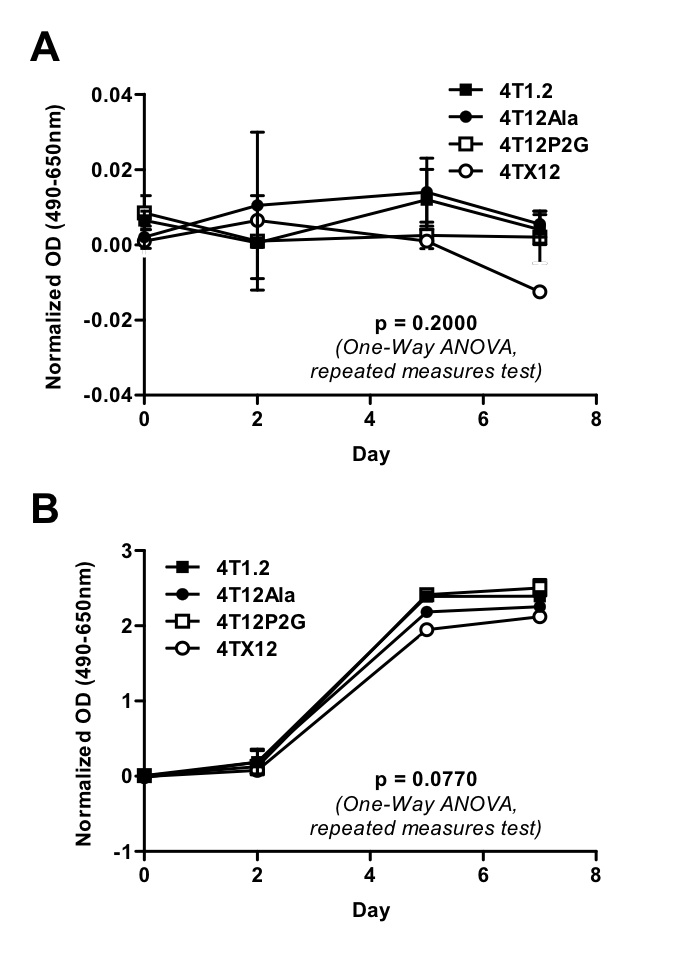

Supplement: Additional file 2 — The effect of CXCL12 expression on in vitro proliferation of 4T1.2 cells in complete medium and serum-reduced medium. Growth of transfected 4T1.2 cell lines as determined by XTT proliferation assay compared to wild-type 4T1.2 cells. (A) Cells grown in medium supplemented with 0.1% FCS. (B) Cells grown in medium supplemented with 10% FCS. Data points represent mean ± SEM of determinations from 2 independent experiments. [file 1476-4598-9-250-S2.JPEG]

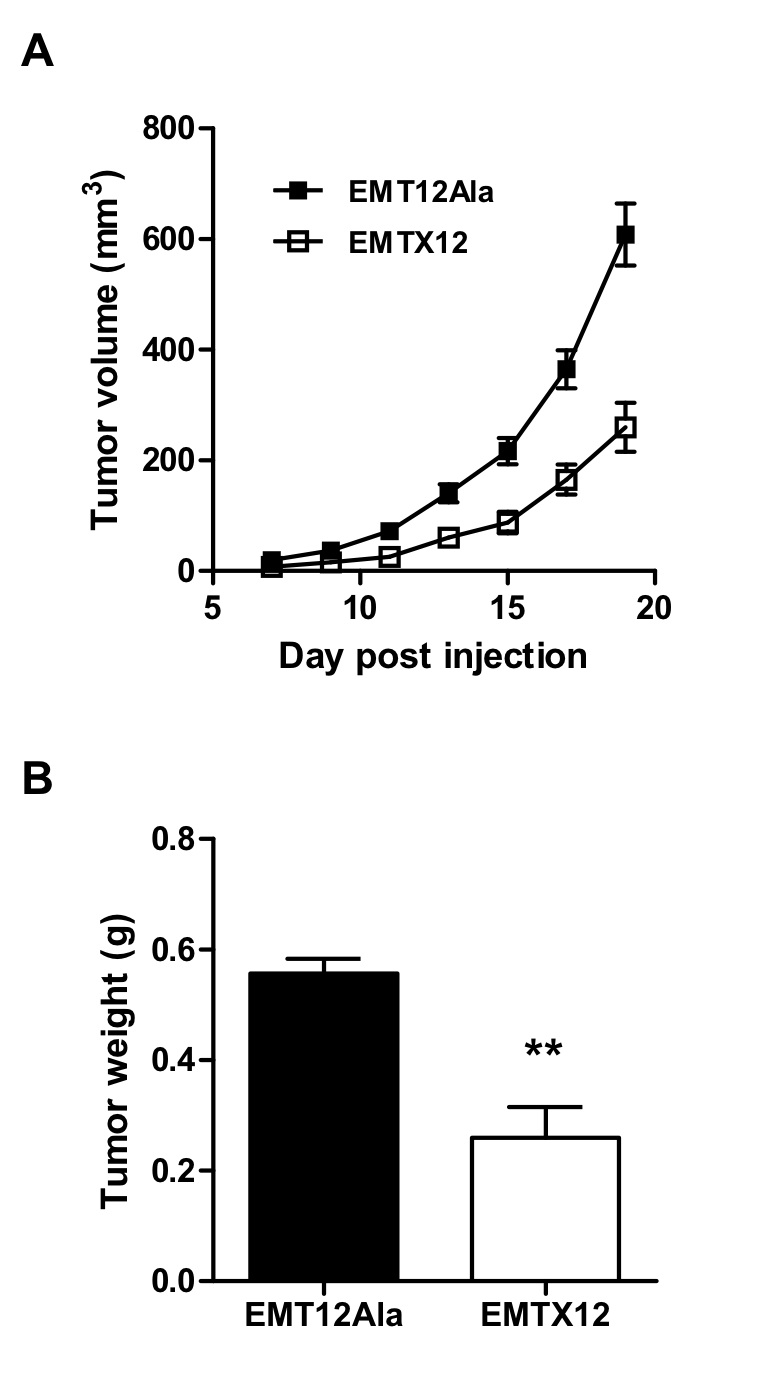

Supplement: Additional file 3 — The effect of CXCL12 expression on growth of EMT6.5 tumors. BALB/c mice were injected with EMTX12 or EMT12Ala cells i.m.f.p. and tumor volume was monitored (A). At the end of the experiment, mice were killed and their tumors resected and weighed (B). **, P < 0.005 (t test), n = 10. Data points and bars represent mean ± SEM. [file 1476-4598-9-250-S3.JPEG]
